# WSIC: a Python package and command-line interface for fast whole slide image conversion

**DOI:** 10.1093/bioadv/vbad122

**Published:** 2023-09-09

**Authors:** Johnathan Pocock, Shan E Ahmed Raza, Fayyaz Minhas, Nasir Rajpoot

**Affiliations:** Department of Computer Science, University of Warwick, West Midlands CV4 7AL, United Kingdom; Department of Computer Science, University of Warwick, West Midlands CV4 7AL, United Kingdom; Department of Computer Science, University of Warwick, West Midlands CV4 7AL, United Kingdom; Department of Computer Science, University of Warwick, West Midlands CV4 7AL, United Kingdom

## Abstract

**Summary:**

Whole slide images (WSIs) are multi-gigapixel images of tissue sections, which are used in digital and computational pathology workflows. WSI datasets are commonly heterogeneous collections of proprietary or niche specialized formats which are challenging to handle. This note describes an open-source Python application for efficiently converting between WSI formats, including common, open, and emerging cloud-friendly formats. WSIC is a software tool that can quickly convert WSI files across various formats. It has a high performance and maintains the resolution metadata of the original images. WSIC is ideal for pre-processing large-scale WSI datasets with different file types.

**Availability and implementation:**

Source code is available on GitHub at https://github.com/John-P/wsic/ under a permissive licence. WSIC is also available as a package on PyPI at https://pypi.org/project/WSIC/.

## 1 Introduction

With the advent of digital slide scanning, several different file formats have been developed to store multi-gigapixel whole slide images (WSIs) produced by digital slide scanners. These file formats are often proprietary or require specialized tooling to handle pixel data and metadata. For example, many WSI formats are a non-standard variant of the Tag Interchange File Format (TIFF). Tiling is an optional extension to the TIFF Revision 6.0 specification (Adobe Developers Association *et al.* 1992) which many image processing tools and libraries either do not support at all or only support with the caveat of decoding all tiles into memory at once. Converting heterogeneous files into a common format may provide predictable performance characteristics, introduce vendor neutrality, and eliminate the need to maintain code dependent on multiple obscure software libraries. However, converting large WSIs can be time-consuming and memory intensive. This is particularly challenging for large WSIs which may be too large to fit into memory. A WSI conversion tool allows interoperability between different vendors’ solutions.

## 2 Features and implementation

We propose a Python application, ‘WSIC’ (Whole Slide Image Conversion), for converting WSIs between various formats. WSIC is designed to be an efficient and scalable tool suitable for converting single files or large data repositories via a command-line interface (CLI). It uses a variety of back-end libraries for reading and writing WSI formats including OpenSlide, OpenJPEG (via glymur), pydicom, wsidicom, tiffile, and zarr/NGFF v0.4 (Moore *et al.* 2021). The application is designed to be scalable and has a memory efficient design and is able to process WSIs in that are too large to fit in memory in a streaming pipeline, much like some other large image processing tools such as libvips (Martinez and Cupitt 2007).

When converting, WSIC uses a multiple reader single writer design pattern. This allows multiple sub-processes to read regions from a source WSI in parallel, while the main process writes decoded image data to the output WSI. This enables the conversion process to scale well on machines with different numbers of CPU cores or amounts of memory.

Additionally, WSIC allows for the region size to differ between the reading and writing processes while handling rearranging the data layout and awaiting required data for writing the next tile. This is useful when converting from formats which are compute intensive to decode, such as JP2 (JPEG 2000). For many JP2 WSIs, it is more computationally efficient to decode fewer large regions than many small regions, thus allowing the read size to be larger than the write size leading to more efficient conversion.

Lastly, when converting between certain formats, it is possible to avoid re-encoding image data. For example, a DICOM WSI may contain many JPEG-encoded tiles which can be directly copied into a target TIFF layout. WSIC can perform this rapid repackaging, referred to as the ‘transcode’ mode in the CLI.

## 3 Conversion benchmarking

To assess performance, we used WSIC 0.8.2 in addition to other open and freely available CLI tools to convert a set of WSIs between a variety of formats. The selected open and freely available CLI tools were: bfconvert 6.12.0 and bioformats2raw 0.5.0 based on the Bio-Formats (Moore *et al.* 2015) library (Java), tiff2jp2 0.1.0 from the glymur (Evans *et al.* 2023) package (Python and C++), and vips 8.13.3 included with the libvips-tools (Martinez and Cupitt 2007) package (C/C++), and Google’s wsi2dicm 1.0.3 (C++) tool (Google Cloud Platform 2007). Graphical user interface (GUI) tools, such as OMERO NGFF-Converter, use the same backend libraries and therefore were not included in the comparison.

Conversion was measured using a desktop computer with a six-core 3.00 GHz Intel Core i5-8500 processor, 64 GB of DDR4 memory, and a solid-state drive. Input formats were JP2, SVS, TIFF, and DICOM. Output formats were JP2, SVS, TIFF, DICOM, and NGFF. NGFF was used as output only because it is a relatively new format where both the format and tooling are still evolving.

A total of 206 unique conversions are performed using five tools and five WSIS, utilizing four possible input and output formats. To account for any potential interference from background system processes, each conversion is performed three times, and the minimum time is recorded. The reported conversion rate in megapixels per second is the average rate across the five images used for each conversion. This method ensures a fair comparison, as the input WSI size may vary.

Except for SVS, where a thumbnail must also be generated, each conversion was performed using only the full high-quality resolution image. This was to reduce confounding factors from the generation of downsamples, where implementations may have involved decisions that trade-off between quality and performance. Furthermore, for most output formats, additional resolutions may be efficiently appended after initial conversion using a chosen downsampling method.

When comparing tools, parameters were normalized across tools where possible. The number of worker sub-processes was set to six (the number of CPU cores available) and the output tile size was fixed at 512 × 512 pixels.

Lastly, a small batch conversion of 20 TCGA SVS images (22.8 GiB in total) across ten tissue types was performed using GNU parallel (Tange 2021) and WSIC ‘transcode’ mode to estimate the time required to batch convert the whole of TCGA (11.8 TiB) using this method.

## 4 Results

Support for reading and writing varied between tools, with WSIC supporting the writing of all formats tested including generic tiled TIFF, SVS, JP2, DICOM (.dcm), and NGFF. BioFormats CLIs, bfconvert and bioformats2raw, also supported writing all formats tested between them. However, half of the cases when reading or writing a JP2 file with bfconvert failed due to an out-of-memory error. The tiff2jp2 tool only supported conversion from TIFF to JP2, while vips only supported writing to TIFF. Additionally, conversions with tiff2jp2 exited with a non-zero exit code and missing tiles were filled by black regions at the bottom and right edges. WSIC preserved the resolution metadata in all tests where the output supported standard resolution metadata, whereas other tools frequently lost this metadata during conversion.

**Figure 1.**
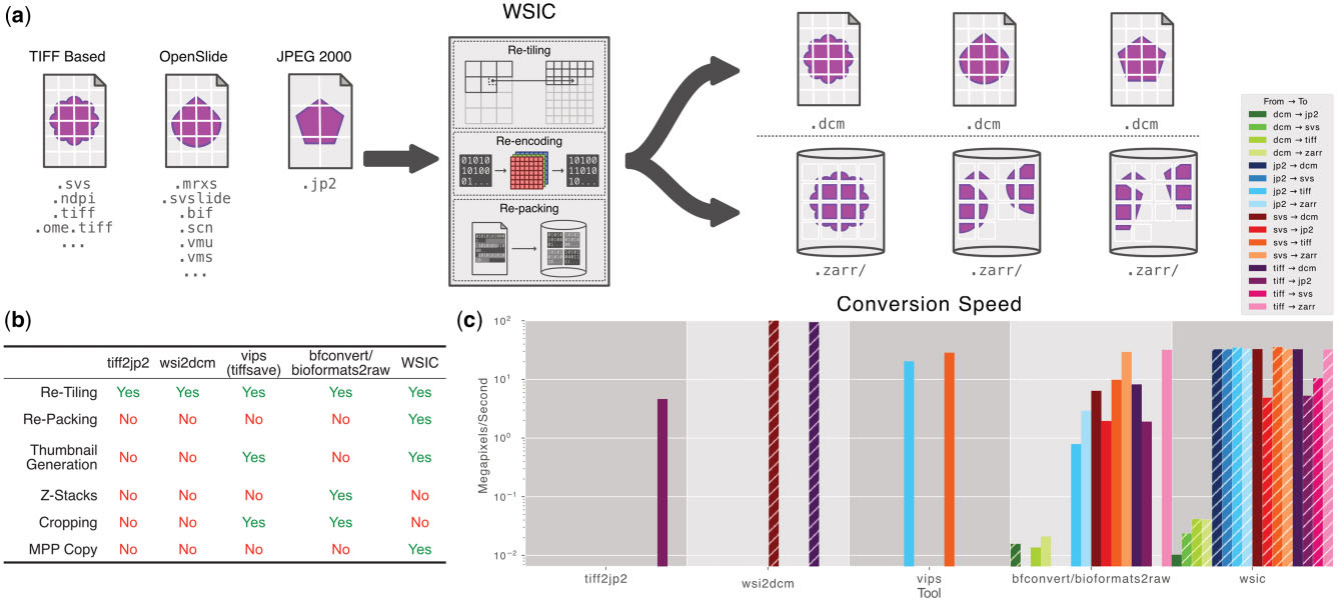
(a) Concept: WSIC can read and write many WSI formats with different codecs and tile sizes. It can convert to cloud-friendly NGFF or the clinical use DICOM format. (b) Feature comparison table. (c) Bar plot: conversion rate shown in megapixels per second for several open-source tools. The highest bar (fastest conversion) for each ‘From → To’ format pair is highlighted with a hashed bar.

Conversion rates for WSIC and other tools are shown in Fig. 1c. The fastest time for conversion was achieved by WSIC in all conversions except for DCM to JP2, SVS to DCM, and TIFF to DCM where bfconvert, wsi2dcm, and wsi2dcm respectively were faster. However, it should be noted that bfconvert failed to write larger JP2 files. Furthermore, WSIC outperformed wsi2dcm in conversion from SVS/TIFF to DCM when the ‘transcode’ mode is used.

Batch SVS transcode for the sample of 20 slides required 135 s in total. Extrapolating this to all diagnostic FFPE slides in TCGA (10.8 TiB) would require 23 h, using the same single desktop computer as in the conversion benchmark.

It’s important to note that the conversion time and resource usage can vary depending on various factors such as the compression codec, tile size, processor, number of threads, or subprocesses used, available memory, disk speed, and image characteristics.

For our test set of typical H&E WSIs and the hardware setup described in the methods section, the following observations were made:

- The average conversion time per WSI with WSIC was 20 s, which is equivalent to a rate of 36 megapixels/s.
- The WSIC transcode mode, which enables rapid repackaging without re-encoding, took an average of 1.4 s per WSI or 445 megapixels/s.
- When using a large read size of 4096 × 4096 pixels, memory consumption for an SVS and JP2 file ranged between 2 GB and 18 GB, respectively.
- Using a smaller read size of 512 × 512 decreases memory usage to between 665 MB and 15 GB at the cost of increased conversion time.
- Decreasing the number of subprocesses can dramatically decrease memory usage. Using only one worker for the JP2 image decreases usage by 1/6 from 15 GB to just 2.5 GB at the cost of a ×6 increase in conversion time.

This profiling was performed with the aforementioned hardware configuration and are subject to change for different hardware configurations.

## 5 Limitations and future work

It’s important to note that the benchmark data presented is specifically for a set of typical WSI images and a specific hardware setup. It’s possible that conversion times may differ on other hardware, datasets, and conversion parameters. For example, images that contain a lot of background may convert more quickly if the codec chosen is sensitive to image entropy. Additionally, there are many settings that can be adjusted to strike a balance between conversion time and resource consumption, such as the number of subprocesses or the size of the region that each subprocess converts at a time.

While this data provides a valuable demonstration of expected performance, it’s important to keep in mind that individual results may vary based on a variety of factors.

This initial version of WSIC targets bright-field WSI images only. This was chosen to limit the initial scope of the project, thereby preventing feature creep and allowing development to focus on the dominant modality of visible light. Visible light or bright-field microscopy such as for Haematoxylin and Eosin and Immunohistochemical stained samples are routinely performed on patients and comprise a large volume of clinical WSI images. Furthermore, standards such as DICOM currently also currently only support visible light microscopy for whole slide imaging and would not be able to be included in this comparison for other modalities.

Future work may expand the range of image formats and modalities supported, such as adding support for multichannel immunofluorescence imaging. WSIC is an open-source project, and we welcome contributions and enhancements from the community via the GitHub repository.

WSIC currently does not copy pyramid levels and their respective data from the source image. This also helped to limit the project scope for version 1.0 and avoid potential issues with corrupted or low-quality reduced resolutions in the source image. An output pyramid with the same pyramid resolutions can be produced by specifying the same downsample factors as in the source image when converting. Planned future work will allow for pyramid levels to be automatically detected and transferred over from the source image. Future work may include an option to copy the pyramid level pixel data instead of recreating them during conversion.

## 6 Conclusion

WSIC shows competitive conversion speed between many WSI formats and offers improved handling of large JP2 images in comparison to other tools, as well as preservation of resolution metadata (microns-per-pixel). In our comprehensive conversion benchmarks, it performed fastest, or near to the fastest performing tool, in every conversion. WSIC also demonstrated suitable speed for the rapid conversion of large-scale conversion of datasets. We expect the digital and computational pathology community to take up this tool and contribute to its further development and expand its use cases.

## Data Availability

The data underlying this application note will be shared on reasonable request to the corresponding author.
